# Report of a Committee to Consider and Report on the Subject of Home Adulterations

**Published:** 1856-04

**Authors:** C. B. Guthrie, Geo. D. Coggeshall, E. S. Wayne, A. J. Matthews

**Affiliations:** Amer. Pharm. Association; Amer. Pharm. Association; Amer. Pharm. Association; Amer. Pharm. Association


### *Report of a Committee to consider and report on the subject of Home Adulterations.*

(From the American Journal of Pharmacy.)

The subject of home adulterations of drugs naturally attracted the attention of the community, and especially of pharmaciéns and physicians, in connexion with that of the foreign, to which we have applied so stringent a law.

No doubt the sophistication of drugs is as well understood in this country as on the other side of the Atlantic, and that if we could apply a remedy as general in its application, we should detect an amount equally astonishing. This is one of the arguments used by the opponents of the drug law, that medicines can be as readily adulterated here as abroad; but we contend that this is no argument against shutting out foreign adulteration, and we hope some of these days to put a stop to the evil at home. The precise method of doing this is not yet apparent, neither is it within the scope of the duties of this Committee to suggest a remedy. One of the results of the different reports from time to time upon this subject, will be to call the attention of the community to the subject, and create a public sentiment that shall demand purer and better medicines when needed, thus drawing the necessary discrimination between the qualities of them when offered either in packages or at retail.

The Committee do not design, at present, a full report, as there are still under their observation and that of others who have aided them in this matter, such articles as are usually met with. Some are of more, some of less importance, all, however, sufficiently so, we think, to merit attention and remark. They are mostly articles that have been found on sale in the interior towns and cities purchased at the cities East, where most of the wholesaling is done. A few instances may be noticed:

*Balsam Peru* has been met with, possessing none of the characteristics of genuine balsam, except in color and consistency, and upon analysis affording no cinnamic acid.

*Pulv. Capsicum.*—The sample examined had a brick dust color, little pungency, and filled with yellow specks and strong odor of *turmeric.* It was a mixture of tumeric and Amèrican capsicum, and, of course, almost inert.

*Castor* is found with the follicles filled with saw dust to half the weight of the castor.

*Opium.*—Since the circular of the Secretary of the Treasury fixing a per centage of morphia for this drug, a more uniform quality has been found in market; but a great many samples have been observed the past season with foreign substances, most commonly lead, inserted in the lumps, in some instances equal to 20 per cent. of the weight of the mass. We are of the opinion that this was done abroad, and probably at the port whence shipped. The different examiners should seek to detect this fraud before passing it.

*Musk* in pod has been observed loaded in the same way, to the amount of 20 grains in a single pod.

*The Essential Oils* are largely adulterated in this country.

*Oil of Peppermint* sometimes contains 50 per cent. of alcohol.

*Oil of Rosemary* is adulterated largely with turpentine, and, in short, the whole class are shamefully sophisticated.

*Otto of Rose* in the same class.

*Cream of Tartar*, adulterated with carbonate of lime, some samples to the extent of 33 per cent., others in less proportion. Sul. potash is also used for this purpose, and alum largely. Of six specimens examined by a gentleman of New York city, purchased at various shops, but one was found pure, some of them being adulterated 30 per cent. The same gentleman says, in reply to our inquiries, that from twenty-two specimens or samples of essential oils, fourteen were found to contain turpentine and other impurities. The same gentleman reports samples of powdered opium adulterated 50 per cent.

*Cod Liver Oil.*—All kinds of fish oil may be found neatly bottled and carefully labelled as the genuine article.

*Sulphate of Quinine.*—Samples have been detected with the old adulteration of mannite, and one gentleman reports quinine mixed up with fine picked raw cotton, adding to the bulk so as to fill the vial without using the requisite quantity of this valuable chemical.

Ipecacuanha in powder and Jalap in powder, each mixed with spurious matter, and English rhubarb in powder, put up for fine powdered Turkey, are not uncommon in all the markets.

Of crude materials, *Nitre*, or Saltpetre is one of the most commonly sophisticated, being adulterated with common salt and nitrate of soda largely.

These are some of the reports made to us, all from reliable sources.

The Committee have endeavored to establish points of observation in different sections of the United States, and as far as possible, to obtain the names of houses from whom these various sophistications have been obtained. Such information they deem it best to withhold from publication at present, lest they might do injustice to parties ignorantly sending out such drugs; but they also intend from time to time to compare notes, and when satisfied of continued practices of this kind, will report such names to the Association.

In the meantime, they cannot too strongly urge retail apothecaries, especially, to be cautious of whom and what quality of medicines they purchase. It is to the dispensing apothecary that medical men and the community look for such medicines as are pure, not only "good of their kind," but of the best kind.

C. B. Guthrie,
Geo. D. Coggeshall,
E. S. Wayne,
A. J. Matthews.

*Proceed. of Amer. Pharm. Association*, 1855.

